# Giant pyogenic granuloma of the thigh: a case report

**DOI:** 10.1186/1752-1947-2-95

**Published:** 2008-03-31

**Authors:** Peter M Nthumba

**Affiliations:** 1 Department of Surgery, AIC Kijabe Hospital, P.O. Box 20 Kijabe 00220, Kenya

## Abstract

**Introduction:**

Pyogenic granuloma or lobular capillary hemangioma remains an etiopathological enigma, with trauma, inflammatory and infectious agents being the commonest suspected causative agents. These lesions affect mucous membranes of the upper aero-digestive tract, and skin. HIV patients diagnosed with pyogenic granuloma present with multiple lesions, caused by *Bartonella spp*.

**Case presentation:**

A 28-year-old woman presented with a solitary large tumor on a skin graft donor site on her left thigh. On excision and histological examination the tumor was found to be a lobular capillary hemangioma (pyogenic granuloma). Further investigation in search of a possible explanation for this unusual presentation revealed HIV infection as the underlying cause.

**Conclusion:**

This report underscores the fact that the full spectrum of presentation of HIV infection is still unknown. Unusual or unexpected presentations should arouse suspicion of underlying immunosuppression, especially in HIV endemic areas.

## Introduction

The term pyogenic granuloma (PG) is a misnomer, and lobular capillary hemangioma is the currently preferred term [1-3]. PG is a benign lesion that occurs on skin and mucosal surfaces of the proximal aero-digestive tracts, but has also been reported to occur subcutaneously, intravenously, in the small bowel, colon and rectum and on burn scars [4-6]. Although cutaneous PG may present as multiple lesions and necrosis, invasion of surrounding tissues is never seen. PG lesions have no malignant potential, but up to 15% may recur following excision [2].

## Case presentation

A 28-year-old female patient presented to AIC Kijabe Hospital (KH) with a large, ulcerated mass on her left thigh which had been present for the previous 6 months.

**Figure 1:**
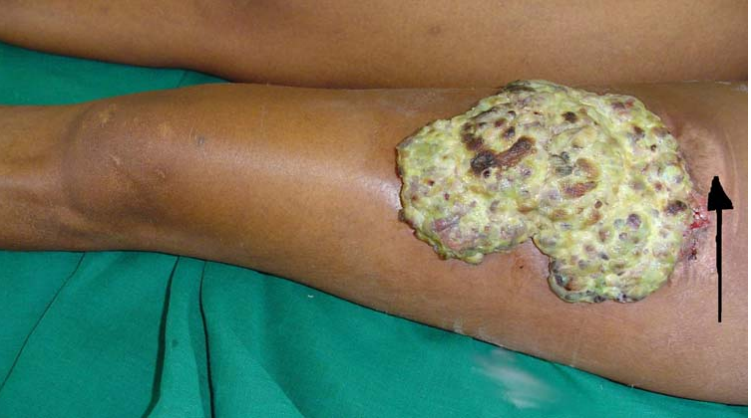
**Giant pyogenic granuloma, left thigh.** Note (arrow) normally healed skin graft donor site on the proximal edge of the mass.

Nine months prior to presentation, she had suffered traumatic avulsion of skin of the anteromedial aspect of her left leg, measuring about 5 cm by 8 cm. The resulting wound became infected and was initially managed with daily dressings. Debridement and split thickness skin grafting (STSG) were finally performed three months after the trauma. The skin graft had been harvested from her left thigh, and whereas the recipient site healed completely, the donor site failed to heal, and instead rapidly developed into a large ulcerated mass with a purulent, foul-smelling discharge. It bled frequently, both spontaneously and on mild trauma. (Figure 1) Severe pain had kept her bedridden for two months prior to her presentation to KH. She had not used any traditional therapies.

The mass was covered by a thick creamy-white exudate. It had a purplish-grey hue around the rolled edges, and measured 25 × 15 × 5 cm. Old blood clots spread across the mass indicated past bleeding episodes. The proximal edge was continuous with the healed skin donor site. She had a healed scar on her leg, and was otherwise in good health.

**Figure 2:**
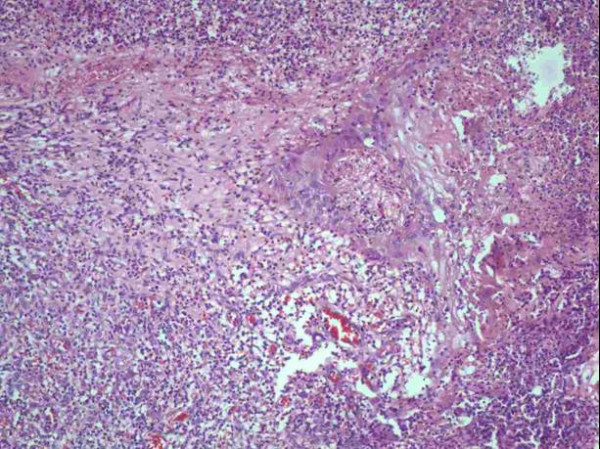
Low power magnification: Note numerous vascular spaces lined by endothelial cells, surrounded by a fibromyxoid stroma with scattered inflammatory cells.

An excision of the mass with a margin of 20 mm was performed, and the defect was covered with a STSG. The biopsy was reported as a giant lobular capillary hemangioma (Figure 2). Bacterial colonization was only present on the surface of the lesion, and there was no evidence of mycobacterial or fungal infection.

**Figure 3:**
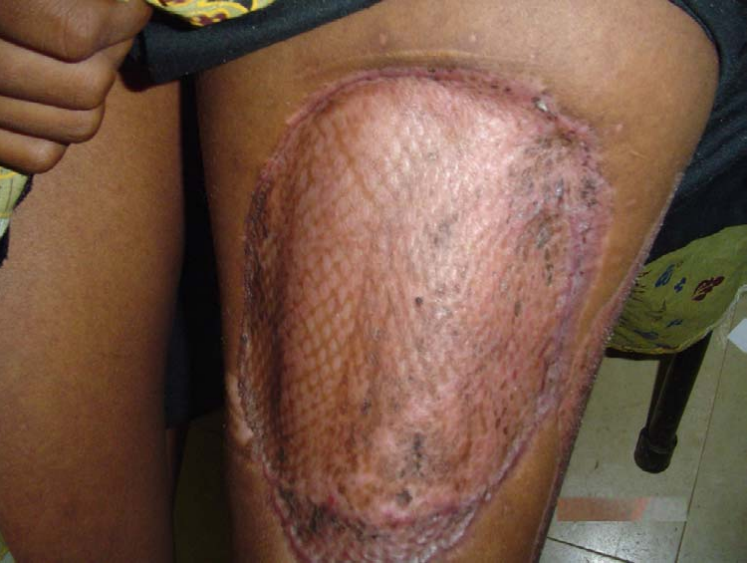
Healed grafted area at 5 months, with no evidence of recurrence.

This unusual history, presentation and diagnosis prompted a search for a possible cause. An ELISA for HIV-1 proved positive. Both the donor and the recipient sites healed completely, with no evidence of recurrence at 5 months (Figure 3).

## Discussion

The cause of PG is not known. However, trauma, hormonal influences, an unknown angiogenic factor; inflammatory and infectious agents, have all been hypothesized as possible factors in causation [2,7]. Mucosal and cutaneous PG appear to be etiologically different, with a higher incidence of mucosal PGs. Many believe that mucosal PGs, which have a higher female preponderance, are causally related to estrogens, while cutaneous PGs are not^9^. Estrogens and other hormones appear to exaggerate the inflammatory responses of gingival tissue, particularly in pregnancy, and lead to the development of PGs in up to 2% of pregnant women (PG gravidarum) [2,8].

Both bacillary angiomatosis (disseminated vascular lesions in immunosuppressed patients), and verruga peruana (crops of vascular nodules in immunocompetent persons), are vascular lesions that resemble PGs, clinically and histologically, and are caused by infection with *Bartonella spp*. Because of this histopathological similarity, some workers have suggested that PG may be caused by *Bartonella spp. *infection [9]. Others have found no association [10,11]. We were unable to exclude *Bartonella spp. *infection as a possible cause in this patient.

## Conclusion

This patient presented with a solitary giant PG on a skin graft donor site, and was found to be HIV positive, an unusual combination, as most HIV-related PGs reported in literature present as multiple lesions, and on staining may show evidence of infection by *Bartonella spp*. [10,12,13].

Most reports suggest that PGs grow to a maximum of 2 cm, save for Choudhary et al. and Tursen et al. who have reported larger lesions [14,15]. Nonetheless, a PG of a size similar to this case has not been reported before.

Skin graft donor sites are known to cause dyspigmentation, hypertrophic scars and keloid. No previous report has been made of a PG arising from a skin graft donor site.

This case report presents a rare manifestation of HIV infection, presenting first as a solitary giant PG, and highlights the wide spectrum of unusual presentations of HIV infection. Thus unusual or unexpected presentations should arouse suspicion of underlying immunosuppression, especially in HIV endemic areas.

## List of abbreviations used

PG – Pyogenic Granuloma; *spp. *– species; STSG: Split Thickness Skin Graft; ELISA – Enzyme-Linked Immunosorbent Assay; HIV – Human Immunodeficiency Virus; AIDS – Acquired Immune Deficiency Syndrome

## Competing interests

There is no association between the author with any commercial firm, and no grants were granted for this article. There are no competing interests in the publication of this article.

## Authors' contributions

The corresponding author came up with the idea, performed the write up and referencing. The author takes sole responsibility of the entire content of this article.

## Consent

Written informed consent was obtained from the patient for the publication of this paper and any accompanying images. A copy of the consent is available for review by the Editor-in-Chief of this journal.
